# Roxadustat for Erythropoiesis-Stimulating Agent Hyporesponsive Anemia in Hemodialysis: Multicenter Retrospective Analysis

**DOI:** 10.3390/medicina62030460

**Published:** 2026-02-28

**Authors:** Ilyas Ozturk, Meliha Ozkutlu, Merve Aktar, Cihan Heybeli, Can Huzmeli, Orhan Ozdemir, Seda Safak Ozturk, Tulin Akagun, Ekrem Kara, Neriman Sila Koc, Mehmet Tuncay, Tuncay Sahutoglu

**Affiliations:** 1 Department of Nephrology, Kahramanmaras Necip Fazil City Hospital, 46050 Kahramanmaras, Türkiye; drilyasozturk@gmail.com (I.O.);; 2 Department of Internal Medicine, University of Health Sciences, Mehmet Akif Inan Education and Research Hospital, 63040 Sanliurfa, Türkiye; 3 Department of Nephrology, Istanbul Aydin University, Medical Park Florya Hospital, 34295 Istanbul, Türkiye; 4 Department of Nephrology, Mus State Hospital, 49100 Mus, Türkiye; 5 Division of Nephrology, Department of Internal Medicine, Faculty of Medicine, Dokuz Eylul University, 35330 Izmir, Türkiye; 6 Department of Nephrology, Hatay Education and Research Hospital, 31027 Hatay, Türkiye; 7 Department of Nephrology, Sanliurfa Education and Research Hospital, 63250 Sanliurfa, Türkiye; 8 Department of Nephrology, Gaziantep City Hospital, 27470 Gaziantep, Türkiye; 9 Division of Nephrology, Department of Internal Medicine, Faculty of Medicine, Giresun University, 28200 Giresun, Türkiye; tulinozbay@yahoo.com; 10 Division of Nephrology, Department of Internal Medicine, Faculty of Medicine, Recep Tayyip Erdogan University, 53100 Rize, Türkiye; 11 Division of Nephrology, Department of Internal Medicine, Faculty of Medicine, Harran University, 63300 Sanliurfa, Türkiye; 12 Division of Nephrology, Department of Internal Medicine, University of Health Sciences, Mehmet Akif Inan Education and Research Hospital, 63040 Sanliurfa, Türkiye

**Keywords:** Anemia, erythropoietin, hypoxia-inducible factor prolyl hydroxylase inhibitors (HIF-PHI)

## Abstract

*Background and Objectives:* Anemia management in maintenance hemodialysis patients with erythropoiesis-stimulating agent (ESA) hyporesponsiveness remains challenging. Roxadustat, a hypoxia-inducible factor prolyl hydroxylase inhibitor, offers a mechanistically distinct alternative. *Materials and Methods:* This multicenter retrospective study analyzed 110 hemodialysis patients with persistent anemia (Hemoglobin (Hb) < 10 g/dL) despite ≥ 3 months of maximum-reimbursable-dose ESA therapy in Türkiye. Outcomes were evaluated between patients who switched to Roxadustat (*n* = 80) and those who continued ESA therapy (*n* = 30) over 6 months in a non-randomized, observational comparison. *Results:* At baseline, median Hb levels were significantly lower in the Roxadustat-group than in the ESA-group (8.70 vs. 9.50 g/dL; *p* < 0.001), while weight-adjusted ESA doses were comparable (*p* = 0.332). By Month 6, the Roxadustat group achieved a significant Hb increase (from 8.70 to 9.95 g/dL), whereas the ESA-group showed no significant change (9.50 to 9.65 g/dL), and end-of-treatment Hb did not differ significantly between groups. The unadjusted mean Hb rise was greater in the Roxadustat cohort than in the ESA cohort (+1.40 ± 1.55 vs. +0.65 ± 1.93 g/dL; *p* = 0.037). However, after adjustment for baseline Hb (ANCOVA), baseline Hb predicted final Hb, while treatment group was not independently associated with final Hb. Transfusion requirements declined over follow-up in both groups. No new short-term safety signal was identified based on available clinical documentation. *Conclusions:* Roxadustat improved Hb in ESA-hyporesponsive patients with lower baseline Hb, but adjusted analyses indicated that baseline severity influenced response. Targets were not consistently achieved; these findings are hypothesis-generating regarding dose optimization, treatment duration, and earlier initiation.

## 1. Introduction

Anemia is a common and burdensome complication in patients undergoing maintenance hemodialysis. Despite the widespread use of parenteral erythropoiesis-stimulating agents (ESAs), there is still a significant number of patients who fail to reach target hemoglobin (Hb) levels, a condition referred to as ESA hyporesponsiveness or resistance. Prevalence estimates for ESA hyporesponsiveness range from 12.5% to 30.3% and are linked to increased cardiovascular risk, diminished quality of life, and elevated healthcare utilization [1,2,3].

ESA hyporesponsiveness in dialysis patients arises from multifactorial causes, including iron deficiency, chronic inflammation, inadequate dialysis, vitamin B12 or folate deficiencies, secondary hyperparathyroidism, and the suppressive effects of uremic toxins on erythropoiesis [4,5]. Central to this pathophysiology is hepcidin—a master regulator of systemic iron homeostasis—whose elevation impairs both intestinal iron absorption and mobilization from stores, reducing the efficacy of iron supplementation and ESA therapy [6,7].

Hypoxia-Inducible Factor Prolyl Hydroxylase Inhibitors (HIF-PHIs), such as Roxadustat, have emerged as mechanistically distinct alternatives. By stabilizing HIF, they enhance endogenous erythropoietin production and suppress hepcidin, thereby improving iron utilization and overcoming inflammation-driven ESA hyporesponsiveness [8].

This retrospective study evaluates the efficacy and safety of Roxadustat monotherapy in maintenance hemodialysis patients with ESA-refractory anemia, defined as persistent Hb < 10 g/dL despite high-dose parenteral ESA therapy.

## 2. Materials and Methods

### 2.1. Study Design and Population

This was a multicenter, retrospective, pragmatic observational study involving maintenance hemodialysis patients from participating nine dialysis centers. Adult patients (≥18 years old) receiving in-center maintenance hemodialysis for at least six months were evaluated for eligibility. Patients were included if they had persistent anemia, defined as Hb levels < 10 g/dL for ≥3 months despite intravenous ESA at the maximum reimbursable dose, capped at 150 U/kg/week epoetin-equivalent by national insurance policy.

Eligible patients were identified retrospectively and classified into two cohorts based on their actual treatment course. Those who had been switched from ESA to Roxadustat during routine clinical care comprised the Roxadustat cohort, while those who continued high-dose ESA despite meeting hyporesponsiveness criteria formed the ESA cohort.

### 2.2. Medication Exposure

In routine clinical practice, concomitant use of ESAs and Roxadustat is not permitted under national reimbursement regulations; therefore, patients who transitioned to Roxadustat did so via an abrupt switch, with ESA discontinued at initiation. Roxadustat was prescribed three times weekly in accordance with approved labeling, and subsequent dose adjustments were made at the discretion of the treating physician as part of usual care.

### 2.3. Data Collection

The study period spanned from June 2023 to June 2024. Within this window, clinical and laboratory data were analyzed at baseline (initiation of treatment) and at a 6-month follow-up point for each patient. Baseline data were defined as the values recorded during the final month of ESA therapy prior to the switch (or the matched index date for controls). Follow-up data were collected at six months post-initiation (±2 weeks). Monthly Hb values were recorded routinely as part of mandated dialysis monitoring protocols.

The following parameters were collected at baseline and follow-up:

- Hematologic and iron indices: Hb, ferritin, serum iron, total iron-binding capacity
- Inflammatory and nutritional markers: C-reactive protein (CRP), serum albumin, uric acid
- Electrolytes and lipids: potassium, low density lipoprotein (LDL)-cholesterol
- Endocrine parameters: intact parathyroid hormone (iPTH), vitamin B12, folate, thyroid-stimulating hormone (TSH)
- Dialysis adequacy: single-pool Kt/V (spKt/V)

Adverse events and complications were recorded if they were documented in the nephrologist’s notes during the routine course of care. These included any thromboembolic events, hypertensive crises, gastrointestinal intolerance, or other significant clinical occurrences. Patients who discontinued treatment, were lost to follow-up, or transferred care were excluded from efficacy analysis but included in descriptive summaries where applicable.

### 2.4. Handling of Missing Data

Due to the retrospective nature of the study and the standardized documentation practices in the dialysis unit, missing data were rare. Data points that were unavailable were not imputed and were excluded from analysis on a per-variable basis.

### 2.5. Study Outcomes

The primary outcome was the change in Hb level (ΔHb) from baseline to six months. Secondary outcomes included treatment tolerability and the incidence of documented clinical complications.

### 2.6. Statistics

Statistical analyses were conducted using JASP version 0.18.1. Continuous variables were presented as mean ± standard deviation (SD) or median (25th–75th), and categorical variables as counts and percentages. Normality of continuous variables was assessed and for normally distributed data, comparisons were made using Student’s *t*-test or analysis of variance (ANOVA); for non-normally distributed data, the Mann–Whitney U test, Wilcoxon signed-rank test, or Kruskal–Wallis test were applied as appropriate.

To evaluate changes in Hb levels over time, repeated measures ANOVA was performed with Greenhouse–Geisser correction due to violation of sphericity. The response to treatment was analyzed using the ΔHb (post-treatment minus baseline value). To assess whether treatment group was associated with final Hb independent of baseline levels, an ANCOVA model was fitted with treatment group as the fixed effect and baseline Hb as a covariate. To further address potential baseline imbalances, an expanded ANCOVA model was subsequently constructed including serum albumin and history of AVF thrombosis as additional covariates. Paired samples were compared using either the paired *t*-test or Wilcoxon signed-rank test, while between-group comparisons were conducted with the independent samples *t*-test or Mann–Whitney U test, depending on data distribution.

**Sensitivity Analyses:** To assess the impact of baseline confounding and selection bias, post hoc sensitivity analyses were performed. Specifically, the ANCOVA model was repeated excluding patients with a history of arteriovenous fistula (AVF) thrombosis and, separately, excluding patients who required blood transfusions at baseline. Post hoc sensitivity analyses were conducted to explore whether extreme baseline vascular morbidity or transfusion burden influenced the observed associations. Effect sizes are reported as partial η^2^ for the primary ANCOVA and η^2^ for sensitivity analyses.

Categorical variables were compared using the chi-square test or Fisher’s exact test, where appropriate. A *p*-value < 0.05 was considered statistically significant in all analyses.

Missing data were minimal (<5%) for all core clinical and laboratory variables, except for selected vitamin, thyroid, and lipid parameters, which were variably available due to routine laboratory testing schedules. Analyses were conducted using available cases (complete-case analysis).

## 3. Results

**Figure 1:**
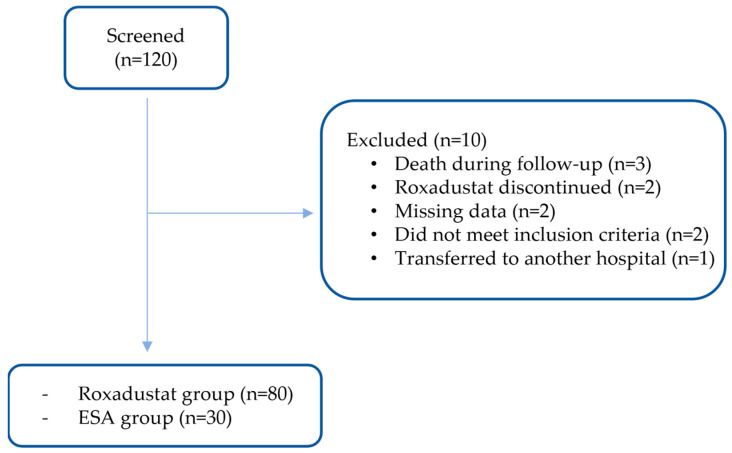
Patient flow diagram of the study cohort. ESA, erythropoiesis-stimulating agent.

Of 120 patients assessed for eligibility, 10 were excluded due to incomplete 6-month follow-up, including death, transfer of care, or missing outcome data (Figure 1). The remaining 110 patients were included in the analysis: 80 transitioned to roxadustat and 30 continued ESA therapy.

### 3.1. Baseline Characteristics

While demographic and dialysis-related characteristics such as age, sex, dialysis vintage, and access type were similar between groups (Table 1), several clinically relevant baseline differences were observed. Patients treated with Roxadustat had higher serum albumin (3.85 vs. 3.20 g/dL; *p* < 0.001), higher ferritin (565 vs. 450 ng/mL; *p* = 0.045), and lower LDL-cholesterol (79.6 vs. 96.2 mg/dL; *p* = 0.027) at baseline. In contrast, a history of AVF thrombosis was markedly more frequent in the ESA group (56.6% vs. 9.5%; *p* < 0.001). Dialysis adequacy remained stable and comparable throughout follow-up (spKt/V ≈ 1.52 at baseline and 1.56–1.57 at Month 6; *p* = 0.793).

### 3.2. Hematologic Response and Iron Parameters

**Figure 2:**
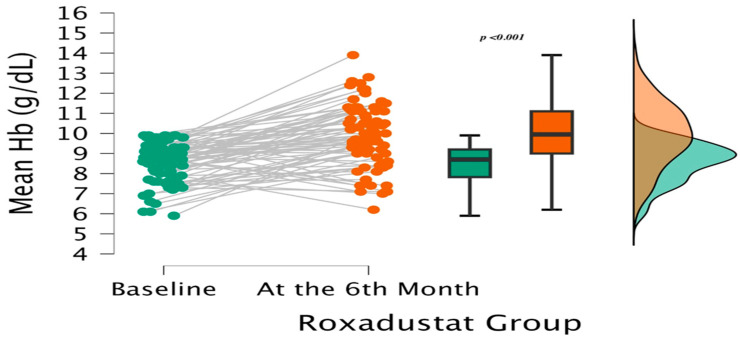
Hb improvement from baseline to Month 6 in the Roxadustat group (ΔHb, *p* < 0.001).

**Figure 3:**
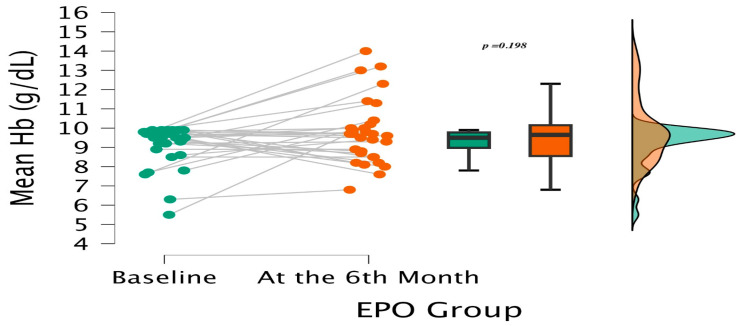
Hb comparison at baseline and Month 6 in the ESA group (non-significant change).

Baseline Hb was lower in the Roxadustat group (8.70 vs. 9.50 g/dL; *p* < 0.001). Over six months, Roxadustat therapy produced a significant rise to 9.95 g/dL (*p* < 0.001 vs. baseline), while the ESA group showed only a modest, non-significant change (to 9.65 g/dL; *p* = 0.198) (Figure 2 and Figure 3).

The mean ΔHb increase was significantly greater with Roxadustat (+1.40 ± 1.55 g/dL vs. +0.65 ± 1.93 g/dL; *p* = 0.037). Although end-of-treatment Hb did not differ statistically between groups (9.95 vs. 9.65 g/dL; *p* = 0.367), the magnitude of Hb improvement was greater in the Roxadustat group.

Given the baseline difference in Hb between groups, an ANCOVA was performed to adjust for baseline Hb levels. In this adjusted model, baseline Hb was a significant predictor of final Hb (F (1,105) = 7.503, *p* = 0.007; partial η^2^ = 0.066), whereas treatment group was not independently associated with final Hb after adjustment (F (1,105) = 1.324, *p* = 0.253; partial η^2^ = 0.012). These findings indicate that baseline anemia severity substantially influenced final Hb levels, attenuating the apparent between-group difference observed in unadjusted analyses.

To further address baseline imbalances, an expanded ANCOVA model was constructed including baseline Hb, serum albumin, and history of AVF thrombosis as covariates. In this model, baseline Hb remained an independent predictor of final Hb (F (1,86) = 6.403, *p* = 0.013; η^2^ = 0.065). The treatment group demonstrated a trend toward association with final Hb (F (1,86) = 3.537, *p* = 0.063; η^2^ = 0.036), whereas serum albumin (*p* = 0.119) and prior AVF thrombosis (*p* = 0.663) were not independently associated. These results suggest that baseline anemia severity remained the primary determinant of follow-up Hb levels, while the treatment effect appeared modest and sensitive to model specification.

Sensitivity analyses were conducted to explore whether baseline vascular morbidity influenced the observed association. In an exploratory subset excluding patients with a history of AVF thrombosis, treatment group was associated with final Hb after adjustment for baseline Hb (ANCOVA: F (1,66) = 4.77, *p* = 0.032; η^2^ = 0.062). Similarly, in an analysis excluding patients who required blood transfusion at baseline, treatment group was associated with final Hb in this exploratory subset analysis (F (1,71) = 4.18, *p* = 0.045; η^2^ = 0.054).

Ferritin remained similar at Month 6 despite higher baseline values in the Roxadustat group. CRP levels were mildly elevated in both arms without significant between-group differences.

### 3.3. ESA Exposure and Roxadustat Dosing

Before treatment transition, ESA use was similar between groups (weekly 9150 [8000–11,550] vs. 9500 [8000–11,500] U/week; *p* = 1). One month prior to therapy change, weight-adjusted ESA doses were also comparable (148.1 vs. 139.4 U/kg/week; *p* = 0.332).

The mean prescribed Roxadustat dose at initiation was 126.8 ± 33.9 mg three times weekly and tapered to 112.7 ± 44.2 mg three times weekly by Month 6. In the ESA group, dose requirements decreased from 139.9 ± 28.1 to 127.25 ± 48.0 U/kg/week (*p* = 0.008).

**Figure 4:**
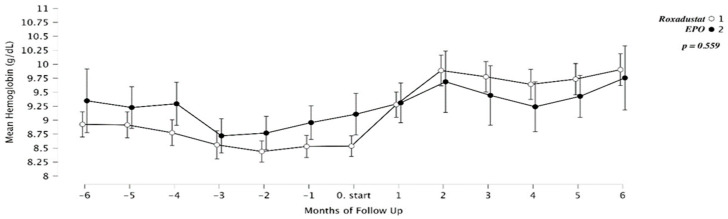
Course of Hb levels during the 6 months preceding the switch to Roxadustat or continuation of high-dose ESA, illustrating persistent ESA hyporesponsiveness.

**Figure 5:**
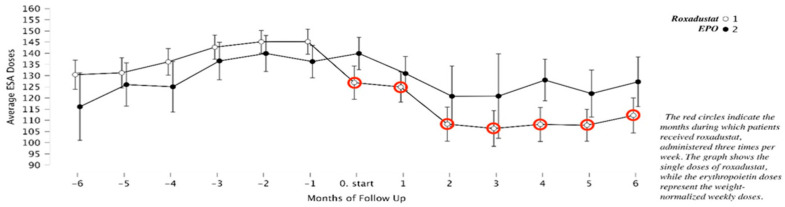
ESA dosing trends during the 6 months preceding the switch to Roxadustat, demonstrating escalating dose requirements without commensurate hematologic response.

Contextualization of ESA hyporesponsiveness: Figure 4 and Figure 5 display Hb and ESA dosing trajectories during the 6 months preceding the switch or continuation decision. These plots contextualize ESA hyporesponsiveness and are not post-treatment outcome data.

### 3.4. Transfusion Requirements and Safety

Transfusion needs decreased in both groups. New thromboembolic events were infrequent and comparable (3 vs. 2 events). Given the significantly higher baseline prevalence of AVF thrombosis in the ESA cohort (56.6% vs. 9.5%; *p* < 0.001), interpretation of thromboembolic risk should consider this imbalance (Table 2). No additional short-term safety signals emerged.

## 4. Discussion

This multicenter retrospective study evaluated the efficacy and safety of Roxadustat monotherapy in maintenance hemodialysis patients with anemia unresponsive to high-dose ESA therapy. Over six months, patients who switched to Roxadustat experienced a significant rise in Hb levels, while those who remained on high-dose ESA therapy showed no significant improvement. Our results suggest that Roxadustat may improve anemia in patients with significant baseline deficits. While statistical models suggest the magnitude of response is proportional to baseline severity—a characteristic shared with ESAs—Roxadustat was associated with clinically meaningful Hb improvement, approaching but not consistently reaching guideline-recommended targets, without the need for continued high-dose ESA therapy. The proportion of patients requiring transfusion declined in both groups, and no increase in adverse events was observed. In adjusted analyses, both in the primary model and in the expanded model including additional baseline covariates, baseline Hb consistently emerged as the dominant determinant of final Hb levels, suggesting that part of the observed improvement may reflect regression to the mean rather than a purely treatment-specific effect. Notably, in sensitivity analyses excluding patients with severe vascular morbidity or baseline transfusion requirements, an independent association between treatment group and final Hb emerged, suggesting that extreme comorbidity burden may obscure treatment effects in unselected real-world cohorts. These sensitivity analyses were exploratory and should be interpreted cautiously, as subgroup restriction may introduce additional instability and reduce statistical precision.

Several studies have investigated the use of Roxadustat in hemodialysis patients with anemia refractory to ESA therapy, examining both monotherapy and combination approaches. Roxadustat monotherapy has shown consistent efficacy across diverse populations. Song et al. (2023) reported that 93.3% of ESA-hyporesponsive patients achieved ≥ 1.0 g/dL increases in Hb, with concurrent improvements in iron metabolism and inflammatory markers [9]. Similarly, Wang et al. (2023) observed significant Hb gains over 12 weeks and identified predictive markers such as platelet-to-lymphocyte ratio and dialysis duration [10]. In peritoneal dialysis patients, Chen et al. (2022) reported that 80% responded to Roxadustat with ≥1.0 g/dL Hb increases, and 50% reached ≥ 11 g/dL by week 12; those reverting to ESA experienced declines [11]. Zhou et al. (2021) also noted target achievement in 48.4% of ESA-hyporesponsive hemodialysis patients [12], and the ROXSTAR Registry demonstrated that over 80% of patients switching from ESA reached Hb targets within 24 weeks [13].

In contrast, limited studies have explored Roxadustat in combination with ESA. Fu et al. (2024) found that dual therapy led to more rapid Hb increases in the first month compared to monotherapy, but the study was small and lacked long-term follow-up [14]. Similarly, Dai et al. (2022) observed that all nine peritoneal dialysis patients receiving combination therapy maintained Hb > 11 g/dL, with two-thirds able to reduce ESA dosage [15]. However, the small sample sizes and limited durations preclude strong conclusions. Notably, the 2025 KDIGO guidelines advise against routine combination therapy due to the absence of randomized controlled trials and concerns over additive thrombotic risk, adverse events, and cost. Compared to these studies, our multicenter cohort reinforces the efficacy of Roxadustat monotherapy in ESA-refractory patients and adds external validity through the inclusion of a concurrent ESA-treated group. Specifically, our analysis clarifies that its clinical utility lies in providing an alternative mechanism for Hb improvement in patients with limited response to high-dose ESA therapy.

Beyond confirming prior reports of hematologic efficacy, our findings highlight the importance of baseline anemia severity in interpreting treatment response in real-world ESA-hyporesponsive populations. The attenuation of the treatment effect after adjustment for baseline hemoglobin suggests that initial anemia burden substantially influences follow-up levels. Importantly, the loss of statistical significance after adjustment does not exclude a treatment effect but indicates that baseline severity explains a substantial proportion of outcome variability. While regression to the mean may contribute in part, the magnitude of hemoglobin increase and the persistence of a treatment signal in selected analyses suggest that pharmacologic and baseline severity effects likely coexist.

ESA hyporesponsiveness in dialysis patients is often driven by chronic inflammation, impaired iron utilization, and elevated hepcidin levels. Inflammatory cytokines interfere with erythropoiesis and stimulate hepatic hepcidin synthesis, which inhibits intestinal iron absorption and macrophage iron release, ultimately leading to functional iron deficiency and reduced ESA efficacy [16,17]. HIF-PHIs, by stabilizing hypoxia-inducible factors, stimulate endogenous erythropoietin production and concurrently suppress hepcidin expression, thereby enhancing iron mobilization and improving erythropoietic efficiency. A recent meta-analysis of 14,737 dialysis patients demonstrated that HIF-PHIs significantly reduced hepcidin levels and intravenous iron requirements while promoting better iron utilization compared to ESA therapy [18]. These properties make HIF-PHIs particularly effective in ESA-hyporesponsive patients, where inflammation-mediated pathways disrupt conventional erythropoiesis.

Despite its efficacy, the FDA declined approval of Roxadustat in 2021 due to safety concerns, including elevated risks of vascular access thrombosis and treatment-related discontinuations. A meta-analysis confirmed a higher rate of vascular complications (RR: 1.15, 95% CI: 1.04–1.27) [19], and another review noted increased withdrawals due to side effects [20]. However, ESA-hyporesponsive patients already face high cardiovascular risk from prolonged exposure to high-dose ESA therapy with poor hematologic response. The ASCEND-D trial reported that such patients had greater ESA requirements, yet limited Hb gains and higher transfusion needs [21]. In this context, switching to Roxadustat may offer a pragmatic alternative and more effective option in selected patients, particularly when prolonged high-dose ESA therapy has failed. In our cohort, no new short-term safety signal emerged; however, interpretation is limited by the retrospective design, modest sample size, and baseline imbalance in AVF thrombosis history. The ROXSTAR registry found that patients transitioning from ESA to Roxadustat achieved target Hb levels without short-term safety concerns, including those previously unresponsive to ESA [13].

This study has several limitations. Its retrospective design, modest sample size, and 6-month follow-up constrain inferences regarding durability of response and long-term safety. Treatment allocation was clinician-driven rather than randomized, resulting in substantial baseline imbalances: the ESA group had a markedly higher prevalence of prior AVF thrombosis and lower serum albumin, while the Roxadustat group exhibited higher ferritin and lower LDL-cholesterol. Although expanded multivariable adjusted analyses incorporating key baseline covariates were performed, statistical adjustment cannot fully substitute for randomized allocation; consequently, residual confounding and confounding by indication remain major concerns that may influence both efficacy and safety signals. Because patients who died, discontinued therapy, or lacked sufficient follow-up were excluded from efficacy analyses, our findings are subject to survivorship bias. This design preferentially reflects outcomes among clinically stable survivors, likely overestimating the magnitude of observed hemoglobin improvement relative to an intention-to-treat framework. Adverse event capture relied on routine clinical documentation and may underestimate event frequency. Furthermore, ESA hyporesponsiveness was defined pragmatically rather than by standardized dose-resistance thresholds. Multiple secondary laboratory parameters, along with exploratory subgroup and sensitivity analyses, were evaluated without formal correction for multiple testing; these findings should be interpreted cautiously as hypothesis-generating, as reduced sample sizes in these subsets may introduce statistical instability. Notably, adjusted analyses indicated that the treatment effect was attenuated when baseline hemoglobin was accounted for, suggesting the observed clinical benefit is primarily driven by the successful management of patients with severe baseline deficits rather than pharmacologic superiority alone. Despite these constraints, this analysis represents one of the few multicenter evaluations of Roxadustat monotherapy in an understudied ESA-hyporesponsive hemodialysis population. The use of a concurrent ESA-treated group and structured outcome assessments strengthens its clinical relevance, providing valuable, hypothesis-generating real-world data.

## 5. Conclusions

Roxadustat monotherapy indicated hematologic improvement in hemodialysis patients with ESA hyporesponsiveness, improving Hb levels in patients with substantial baseline deficits. While the magnitude of the Hb rise was influenced by baseline anemia severity, the treatment was associated with improved Hb levels compared with continued high-dose ESA therapy in unadjusted analyses. Transfusion requirements decreased over follow-up, though between-group differences at follow-up were not statistically significant. No imbalance in observed short-term adverse events was detected, acknowledging retrospective event capture. These real-world findings suggest Roxadustat as a potential alternative for managing anemia in this high-risk population. However, the median Hb remained below 10 g/dL at six months, underscoring the need to refine dosing strategies and patient selection. Long-term safety and durability of response require confirmation in prospective, controlled studies.

## Figures and Tables

**Table 1:** Baseline and 6-Month Characteristics ^†^.

Variable	Baseline		*p*	Month 6		*p*
	ESA (*n* = 30)	Roxadustat (*n* = 80)		ESA (*n* = 30)	Roxadustat (*n* = 80)	
Demographics						
Age (y)	50.9 ± 15.3	52.6 ± 14.9	0.611	—	—	—
Male/Female	12/18	36/44	0.638	—	—	—
HD vintage (mo)	56 (18–86)	45 (28.2–62.2)	0.414	—	—	—
Dry weight (kg)	70.7 ± 13.8	65.5 ± 15.8	0.116	—	—	—
Vascular access						
AVF, *n* (%)	22 (73)	65 (81)	0.481	—	—	—
Cuffed catheter, *n* (%)	8 (27)	14 (17.5)		—	—	—
Uncuffed catheter, *n* (%)	0	1 (1.2)		—	—	—
Residual renal function *						
Residual function, *n* (%)	6 (20)	22 (27.5)	0.457	—	—	—
Comorbidities/habits						
Smoking, *n* (%)	6 (38)	11 (20)	0.138	—	—	—
COPD, *n* (%)	2 (6.7)	6 (7.5)	0.881	—	—	—
Hypertension, *n* (%)	26 (87)	57 (71)	0.094	—	—	—
Heart failure, *n* (%)	4 (13)	10 (13)	0.907	—	—	—
CAD, *n* (%)	8 (27)	13 (23)	0.722	—	—	—
Stroke, *n* (%)	3 (10)	12 (15)	0.496	—	—	—
Diabetes, *n* (%)	14 (47)	27 (34)	0.212	—	—	—
AVF thrombosis history, *n* (%)	17 (56.6)	6 (9.5)	<0.001	—	—	—
Dialysis dose						
spKt/V	1.52 ± 0.25	1.52 ± 0.31	0.993	1.56 (1.36–1.76)	1.57 (1.40–1.76)	0.793
Key laboratory indices						
Hb (g/dL)	9.50 (8.67–9.77)	8.70 (7.87–9.20)	<0.001	9.65 (8.55–10.15)	9.95 (9.00–11.10)	0.367
Uric acid (mg/dL)	6.00 ± 1.54	6.80 ± 1.49	0.015	—	—	—
Potassium (mEq/L)	4.77 ± 0.68	5.18 ± 0.92	0.030	—	—	—
Albumin (g/dL)	3.20 (3.02–3.50)	3.85 (3.57–4.10)	<0.001	—	—	—
intact PTH (pg/mL)	636 (371–1100)	474 (258–880)	0.115			
TSH (mIU/L) (*n* = 41)	1.34 (1.09–1.70)	1.60 (1.01–2.35)	0.487			
B12 (pg/mL) (*n* = 85)	375 (315–584)	487 (322–683)	0.309			
Folate (ng/mL) (*n* = 81)	3.74 (3.15–4.31)	7.90 (5.40–11.10)	<0.001			
Ferritin (ng/mL)	450 (320–605)	565 (422–826)	0.045	590 (375–826)	553 (371–821)	0.595
LDL-C (mg/dL) (*n* = 77)	96.2 ± 27.9	79.6 ± 32.4	0.027	—	—	—
CRP (mg/L) (*n* = 97)	10 (4.2–20.5)	8 (4.5–16)	0.643	6.5 (3–15.7)	10 (5–17)	0.211
ESA dose (U/kg/week)	139.4 (112–160)	148.1 (134–153)	0.332	—	—	—

^†^ Variables with lower availability are shown with sample sizes; others had >95% completeness. Missing data were handled by complete-case analysis. Data are presented as mean ± SD, median (25th–75th percentiles), or *n* (%), as appropriate. Abbreviations: AVF, arteriovenous fistula; CAD, coronary artery disease; COPD, chronic obstructive pulmonary disease; CRP, C-reactive protein; ESA, erythropoiesis-stimulating agent; Hb, Hemoglobin; HD, hemodialysis; LDL-C, low-density lipoprotein cholesterol; PTH, parathyroid hormone; spKt/V, single-pool Kt/V; TIBC, total iron-binding capacity; TSH, thyroid stimulating hormone; B12: Vitamin B-12. * Residual renal function: Preserved native kidney function reflected by measurable urine output (≥100 mL/day) in dialysis patients, representing the remaining glomerular filtration capacity.

**Table 2:** Transfusion and safety outcomes (Roxadustat vs. ESA groups).

Parameter	Roxadustat (*n* = 80)	ESA (*n* = 30)	*p*-Value
Transfusions			
Patients requiring transfusion, baseline	37.5%	26.7%	0.037
Patients requiring transfusion, follow-up	27.5%	13.3%	0.119
Thromboembolic events			
History of AVF thrombosis, baseline	9.5%	56.6%	<0.001
New thromboembolic events during follow-up, *n*	3	2	NR
Other safety findings	No new short-term safety signal was identified in recorded clinical documentation.		

## Data Availability

The data presented in this study are available on request from the corresponding author. Data are not publicly available due to legal and ethical reasons.

## Abbreviations

The following abbreviations are used in this manuscript:

ESA	Erythropoiesis-stimulating agent
Hb	Hemoglobin
HIF-PHI	Hypoxia-Inducible Factor Prolyl Hydroxylase Inhibitors
CRP	C-reactive protein
LDL	Low density lipoprotein
iPTH	Intact parathyroid hormone
TSH	Thyroid-stimulating hormone
spKt/V	Single-pool Kt/V
ΔHb	Change in Hb level
SD	Standard deviation
AVF	Arteriovenous fistula
CAD	Coronary artery disease
COPD	Chronic obstructive pulmonary disease
HD	Hemodialysis
TIBC	Total iron-binding capacity
KDIGO	Kidney Disease Improving Global Outcomes
FDA	U.S. Food and Drug Administration
